# Nanoscopically Confined 1,4-Polybutadiene Melts: Exploring Confinement by Alumina Nanorod and Nanopore Systems

**DOI:** 10.1021/acs.jpcb.4c04553

**Published:** 2024-10-15

**Authors:** L. Tannoury, W. Paul

**Affiliations:** Institüt für Physik, Martin-Luther-Universität, D-06099 Halle, Germany

## Abstract

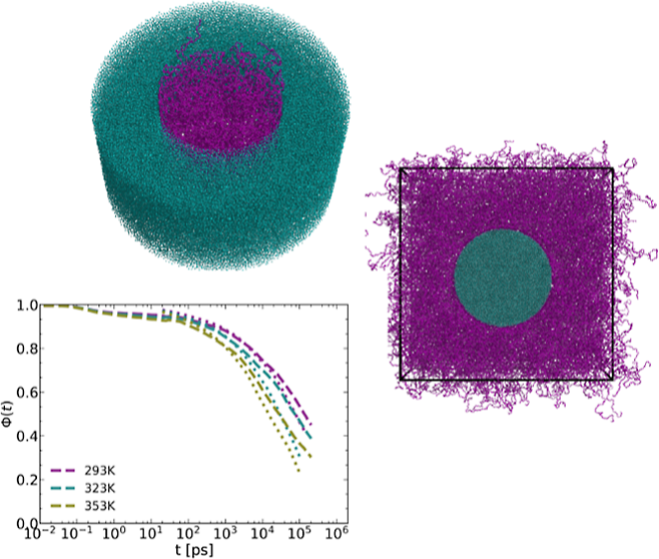

We present molecular dynamics simulations of a chemically realistic model of 1,4-polybutadiene (PBD) in contact with curved alumina surfaces. We contrast the behavior of PBD infiltrated into alumina pores with a curvature radius of about three times the radius of gyration of the chains to its behavior next to a melt dispersed alumina rod of equal absolute curvature. These confinement types represent situations occurring in polymer melts loaded with nanoparticles due to nanoparticle aggregation. While there are observable differences in structure and dynamics due to the different types of geometric confinement, the main effects stem from the strong attraction of PBD to the alumina surfaces. This strong attraction leads to a deformation of the chains in contact to the surfaces. We focus on temperatures well above the bulk glass transition temperature, but even at these high temperatures, the layers next to the alumina surfaces show glass-like relaxation behavior. We analyze the signature of this glassy behavior for neutron scattering or nuclear magnetic resonances experiments.

## Introduction

Polymer nanocomposites (PNC) have a wide range of applications in modern high-tech industries. They are made of nanosized particles incorporated into a polymer matrix aiming for an improvement of the properties (mostly its mechanical properties) of the composite material compared to the pure polymer. The improvement of the macroscopic properties of the PNC is generated by the existence of an interface around the fillers in which structure and dynamics of the polymers are altered. Nanofillers create a large amount of such interface for a fixed filler volume fraction leading to strong effects. The change in mechanical behavior of this interface region, which typically has a thickness of a few (or a few tens) of nano meters, is governed by a change of the glass transition behavior in this region. The confinement alters the molecular properties of the adjacent polymer material and with this its glass transition behavior. A molecular understanding of the structural and dynamical changes in the interface would allow for a design of specific macroscopic properties of a composite material.^1^ This goal has drawn a lot of research interest to the subject.^1−11^

Beyond the material science interest, however, these systems are of fundamental interest with regard to the statistical thermodynamics of confined matter. Sufficiently strong interaction with a surface can induce ordering phenomena (surface induced ordering), for instance in magnetic systems,^12^ but also for liquids, manifesting itself in wetting phenomena.^13^ Recently, even a surface induced crystallization of a polymer melt has been identified.^14^ Such surface induced ordering might play a role in composite materials of semicrystalline polymers as well. But even for the amorphous phase the interaction with a surface can have strong structural consequences, which are most clearly brought out by the phenomenon of ultrastable glasses.^15,16^ These amorphous systems reach much higher density than bulk-cooled glasses and in the case of organic molecules, for which they were found first, may also exhibit some local orientation order.^17^

Over the past couple of decades, several theoretical studies,^18−21^ coarse-grained^22−36^ and atomistic simulations^37^ have been conducted to predict and investigate the structure and conformation of PNC. For spherical nanoparticles (NP), some theoretical studies^19,20^ and coarse-grained simulations^32,38^ found a density reduction at the NP surface for sufficiently weak interaction with the polymer. Typically, however, the attraction is sufficiently strong^25,31,37,39−42^ to lead to a density increase and layering effect at the NP surface. For attractive spherical NP smaller than the polymer chains,^18^ also the polymer chain dimensions may be perturbed.

Experimental studies have tried to create a macroscopic mechanical response by introducing NP in a controlled manner.^43−45^ Several studies also addressed the local molecular dynamics of polymers chains in nanocomposites.^23,25,33,34,46−52^ They typically find a decrease in mobility of the monomers as they approach the surface of the confinement. There is a slowing down of the dynamics in the layer adsorbed to the confinement walls until bulk-like behavior is obtained as one moves away from the surface.

PNC typically exhibit a fractal-like aggregation of the NP (see, e.g., the work^44^). In this environment, polymer chains are exposed to convex and concave surfaces of varying curvature. It is unclear still, to which degree the structural and dynamical response of polymer chains in the interface depend on the type of curvature. We will therefore study this for a random copolymer of 1,4-polybutadiene (PBD) adsorbed in a pore of alumina on the one hand, and in contact with an alumina rod of the same absolute curvature on the other hand.

The interaction with the surfaces of the nanoconfinement introduce an additional energy scale and it has been shown by simulations that this interaction leads to an additional relaxation process, the desorption of polymer chains from the surface, whose time scale can be much larger than bulk relaxation time scales.^53−55^ We will study this desorption process here for the PBD-alumina interface for the pore and the rod case.

## Model and Simulation Method

We will be studying a united atom model of PBD random copolymers with 55% trans and 45% cis monomers (i.e., neglecting the possibility of vinyl monomers). The CH_2_ and CH united atoms differ by mass and Lennard-Jones interaction parameters, the CH_3_ groups only differ from the CH_2_ ones by their mass. The chains consist of 29 repeat units, i.e., *N* = 116 united atoms along the backbone. This chain length still shows Rouse behavior. The force field was developed in ref (56) and validated for bulk simulations.^57,58^ PBD is an almost apolar polymer with very small partial charges on the cis group which were neglected in the dynamical simulations of the bulk behavior which were able to quantitatively predict experimental data. We therefore chose not to include them for the confinement simulations, also.

The alumina force field was taken from the literature^59^ and an amorphous bulk sample was created following the procedure discussed in detail in ref (60). Into this sample a cylindrical pore of 10 nm diameter was cut and infiltrated with a PBD melt.^60^ From the same sample we also cut an alumina rod of 10 nm diameter and equilibrated a polymer melt around it. The number of chains for both systems were chosen such that the melt density away from the respective surfaces are close to the bulk density of PBD at the simulation temperatures. For the pore systems, we used 275 chains and for the rod systems 1361 chains in a cubic simulation box of size *V* = 21.55 × 21.55 × 10.602 nm^3^, the latter being the length of the nanorod. The radius of gyration of the chains in the bulk is 1.5 nm, so the ratio of gyration radius to pore respectively rod radius is around 3.3. We are therefore in a region of moderate confinement. This ratio is fixed by the smallest size of alumina tube one typically creates experimentally and the chain length studied before extensively in the bulk. To go to strong confinement (radius of gyration of size equal or smaller to the pore radius) would necessitate the simulation of deeply entangled PBD melts in confinement, which is beyond the capabilities of atomistic simulations.

As we are neglecting the partial charges on the PBD, we also do not take into account the charges on the aluminum and oxygen atoms in the alumina, i.e., the interaction between PBD united atoms and the atoms in the alumina are purely of Lennard-Jones type. This underestimates the strength of the attraction to this surface, which may be rather strong according to a recent comprehensive study of *cis*-1,4-polybutadiene at a flat crystalline alumina wall.^61−63^ The authors performed a density functional theory study^61^ on the interaction between adsorbed PBD monomers and the alumina wall atoms and found strong electronic correlation effects. To take these into account in a simulation would require an ab initio approach, which would, however, be prohibitive for the time scales of relevance. The authors found a clever way to circumvent this by abandoning the Lorentz–Berthelot combining rules for the dispersive interaction and using a short-ranged and very strong Lennard-Jones interaction between the PBD united atoms and the wall atoms (the Lennard-Jones interaction between the aluminum atom and the sp^2^ carbon, for example, has a minimum at a distance of around 2 Å from the surface with a depth of about 5.6 kcal/mol, i.e., approximately 3000 K^61^). Consequently, the first atom layer at the wall has about a decade lower mobility then the next layer, as judged by an effective subpicosecond diffusion coefficient^63^ (Figure 2b). An attraction strength of around 3000 K to the surface, however, basically prohibits exchange between the wall layer and the bulk in the simulation at all temperatures of interest. While this may be the true situation at an alumina substrate, it generates problems of statistics for the small simulation boxes available to the MD approach. Also, we want to study the effect of concave vs convex confinement and this will only become relevant, if the chains manage to desorb from the surface within the time scale of the simulation. We therefore chose to work with the standard Lennard-Jones interactions only. We will see that also then the attraction to the alumina surface already is strong enough to induce relevant structural and dynamic effects. In contrast to the studies^61−63^ we work with an amorphous alumina confinement as realized in the experimental situation of alumina pores. We do not consider the possibility of surface reconstruction of the alumina or the adsorption of water onto this surface.

We performed simulations at three temperatures, *T* = 353, 323, and 293 K where PBD is a melt. At these temperatures, no indications of the glass transition in this polymer are yet visible for bulk simulations, so all glass-like relaxation processes we find will be purely surface induced. The simulation runs covered 220–410 ns, depending on temperature and system. Prior to the production runs, we took 30 ns as equilibration time, which is more than enough to guarantee bulk equilibration at these temperatures.

## Results and Discussion

### Structure of the Melt

**Figure 1:**
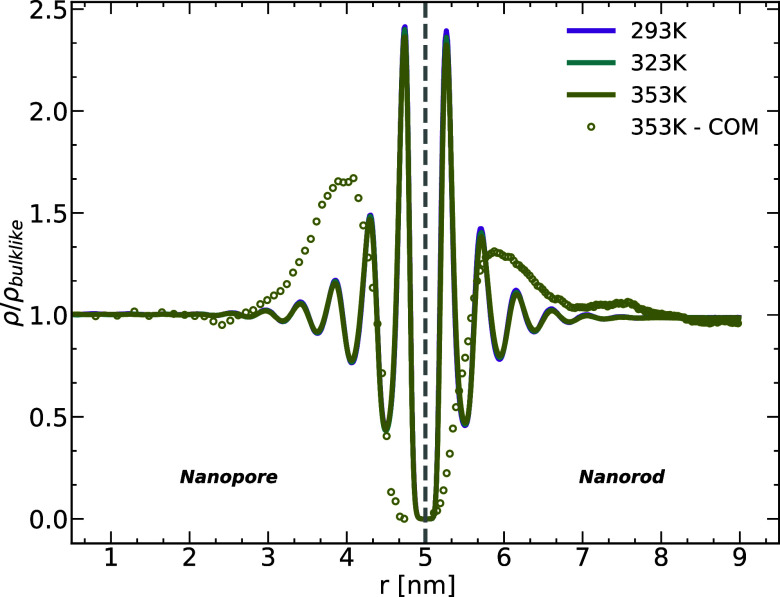
Monomer and chain COM (*T* = 353 K) density profiles of the PBD melt for the pore (left) and rod (right) systems. *r* is the radial position with a value of ∼5 at the pore/rod walls. *r* → 0 is toward the center of the nanopore and *r* → 9 is moving away from the nanorod. The curves for all three temperatures (as well as the COM density) are normalized to their value away from the confinement walls. The volume of the bins is kept constant.

In the following section, we will study the impact of the presence of alumina surfaces on the structure of the PBD melt, exploring the potential impacts of curvature and confinement across several scales. A well documented phenomenon in the study of melts under confinement is the density layering at the interface as a result of the atomic interactions between the melt and the substrate. Figure 1 shows the monomer density (for all three temperatures *T* = 293, 323, and 353 K) along the radial direction, , in both the pore and rod systems, along with the center of mass (COM) density for *T* = 353 *K*.

**Figure 2:**
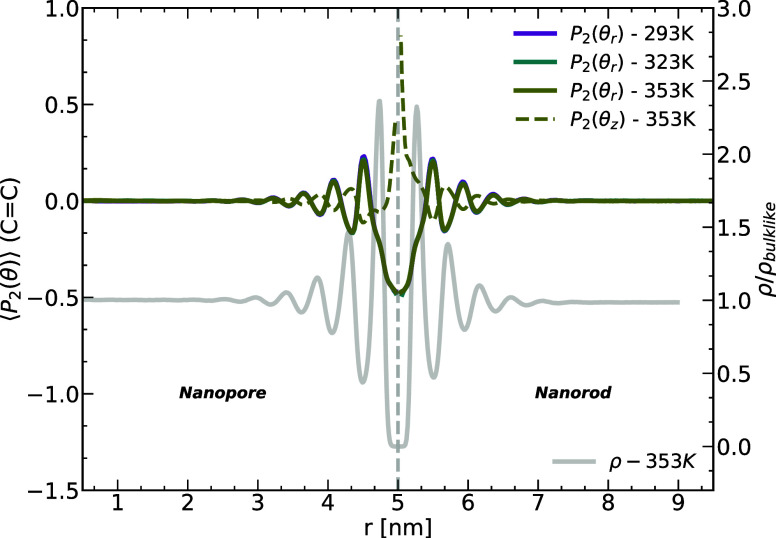
Orientation ordering of the double bonds in the chains units given by the second Legendre polynomial of the angle between the double bonds and the radial (solid lines) and axial (dashed lines for *T* = 353 K) directions. The position is determined by the radial position of the double bonds’ centers of mass. The gray line is the density profile at *T* = 353 K (right *y*-axis).

In this figure and the all the figures in the current section, *r* in the plots is the radial position with a value of 5 at the walls which increases or decreases as we move away from the nanorod and nanopore surface, respectively. The density curves are all normalized to their value away from the alumina walls and the volume of the bins around a position, *r*, for which the densities are calculated, are kept constant throughout the melt. The density profiles have a well-defined layered structure with a distinct enhancement in the first layers as a consequence of the melt-alumina attraction. Both systems exhibit a length scale of the monomer size (∼σ) in the layer distances. The perturbations extend to ∼2.5 nm away from the alumina walls after which the melt reaches a density comparable to that of the bulk at *T* = 353 K (865 kg/m^3^) within a ∼2.1% (pore) and ∼0.58% (rod) percentage difference. The layering for the COM density has a length scale of the radius of gyration of the PBD chains *R*_g_ ≈ 1.5 nm. The similarity of the density profiles for both systems indicates that for a curvature radius sufficiently larger than the monomer diameter, the density profile is mostly affected by the type of interaction between the melt and the surface. The slight difference that can be seen in the amplitudes of the peaks can be related to the fact that the volume accessible for the monomers in the first layer at the wall is slightly smaller for the nanopore system than its nanorod counterpart. The excess density contained within the first 2.5 nm from the walls defines an interfacial layer. To examine the orientation ordering of the segments in that layer and compare it to the rest of the melt, we calculate the second Legendre polynomial of the angle θ
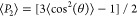
1where θ is defined as the angle between the double bonds and  and , respectively. Figure 2 shows ⟨*P*_2_⟩ for all three temperatures for the angle θ_*r*_ and for *T* = 353 K for θ_*z*_ for both systems (nanopore and nanorod) as well as the density profile at *T* = 353 K as the gray solid line. ⟨*P*_2_⟩ has an average value of −0.5 or 1 if the double bonds are perpendicular or parallel, respectively, to the studied direction. Similar to the density profile, the oscillations of the double bond order parameter extend to around 2.5 nm away from both alumina walls. The solid curves, which represent ⟨*P*_2_⟩ for θ between the double bonds and the normal to the alumina surface , show comparable results for both systems. These results indicate that the double bonds prefer to orient themselves perpendicular to the surface normal vector and along the alumina walls for both curvatures. ⟨*P*_2_(θ_*z*_)⟩; however, reveals that for the nanorod system, the double bonds tend to be more aligned with the axis of the cylindrical confinement with a value very close to 1. Similar findings have also been reported by Patsalidis et al.^62^

The structure and ordering on the chain scale can be accessed by studying their gyration tensor
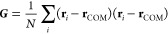
2

**Figure 3:**
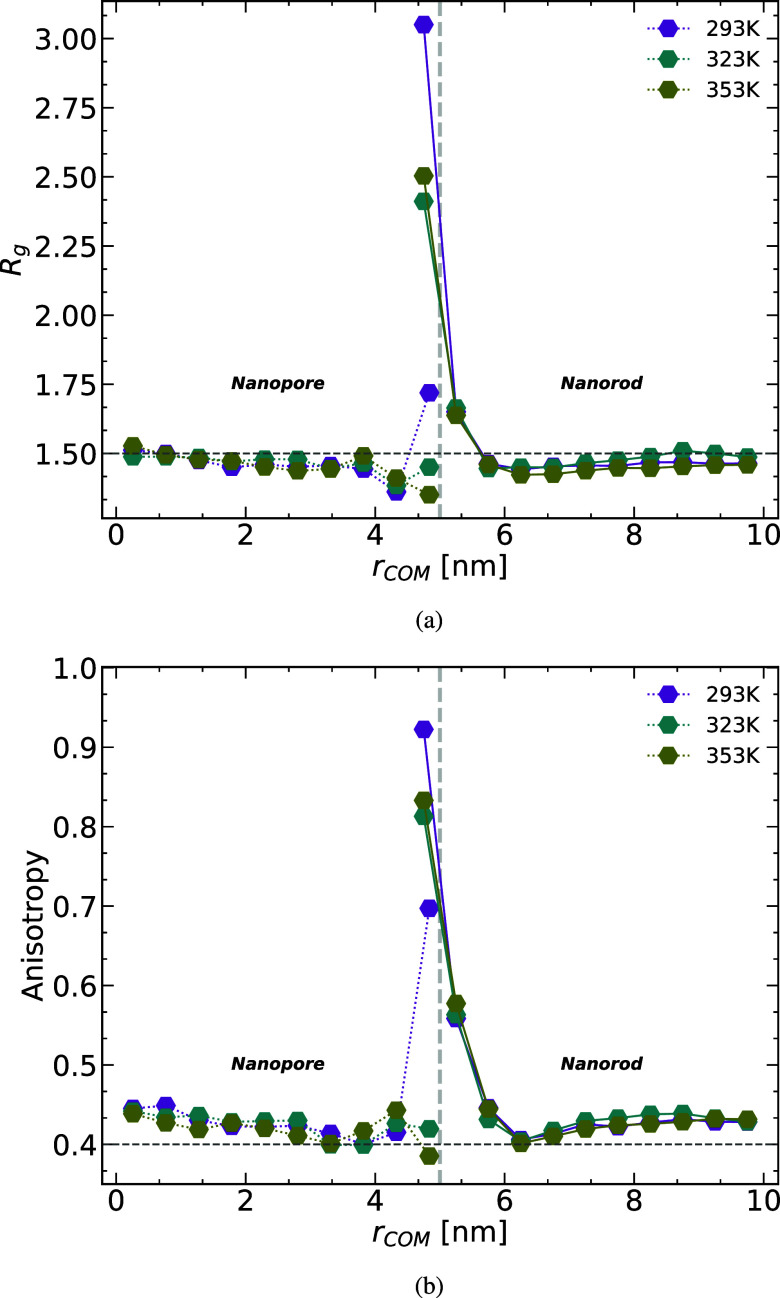
Comparison between the rod and pore systems of the values of *R*_g_ (a) and the chain anisotropy (b) for all three temperatures.

The radius of gyration of the chains, *R*_g_, is obtained from the trace of the gyration tensor . For the confined PBD melt the chains’ gyration radius is close to its bulk value (horizontal gray line) except at the pore/rod surface as can be seen in Figure 3a. For the pore, the value of the radius of gyration in the first layer starts to increase at the lowest temperature, for the nanorod system it is for all temperatures much larger than the bulk value, reaching two times the bulk value at the lowest temperature. These findings indicate that the chains are significantly deformed upon adsorption onto the alumina surfaces, contrary to what was found for a graphite confinement, where they were only oriented due to the much weaker attraction in that case. A good measure for the deformation of the chains is the relative shape anisotropy κ^2^ (seen in Figure 3b) that can be calculated from the eigenvalues of the gyration tensor as given in eq 3
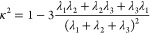
3κ^2^ describes the symmetry and dimensionality of the chains and takes a value of zero for spherically symmetric conformations and 1 for rod-like chains. The value of around 0.42 in the bulk is typical for the gyration ellipsoid of a bulk melt chain. It is important to note that for the rod geometry, if a chain is wrapped around the nanorod then its COM position can be at a radial position smaller than 5 nm (radius of the rod). According to our simulations, only a few chain centers of mass are in that layer throughout the whole trajectory; however, they reach a basically rod-like conformation with twice the bulk radius of gyration at *T* = 293 K.

In addition to the size of the chain, studying the eigenvectors of the largest eigenvalues of the gyration tensor, λ_1_, presents us with information about the orientation of the chains. The curvature effects in the nanopore system produce an alignment of the chains with the axis of the cylindrical pore. The few chains directly situated at the wall layer in the rod case tend to align along either the  or  axes, with minimal contribution from the axial direction. Consequently, they exhibit a preference for alignment perpendicular to the  axis. Moving beyond this region, approximately 1 nm away from the rod surface, there is an observable shift in the preferred alignment of the chains toward the axial direction. Subsequently, these chains exhibit bulk-like behavior, resembling their counterparts studied at the central region for the pore case. Previous studies on polymer melts with NP^25,64^ have demonstrated similar behaviors where the chains in the first layer at the interface are “flattened” against the nanoparticle. The outcomes from both systems indicate that the ordering, conformation, and orientation of the chains are influenced not only by the interaction between the melt and the confinement but also by the curvature of the latter.

### Dynamics

#### Dynamics on the Chain Scale

The structural changes in the polymer melt close to the confining surfaces also lead to changes in the relaxation behavior. We start our analysis with the mean squared displacement (MSD) of the chain COM. We have discussed the COM MSD of the pore system in a previous publication^60^ and focus here on the rod case and a comparison between the two confining geometries. We divide the melt into three different layers each of size ∼*R*_g_ of the PBD chains. A chain belongs to a certain layer if the radial position of its COM is in that layer at *t* = 0.

**Figure 4:**
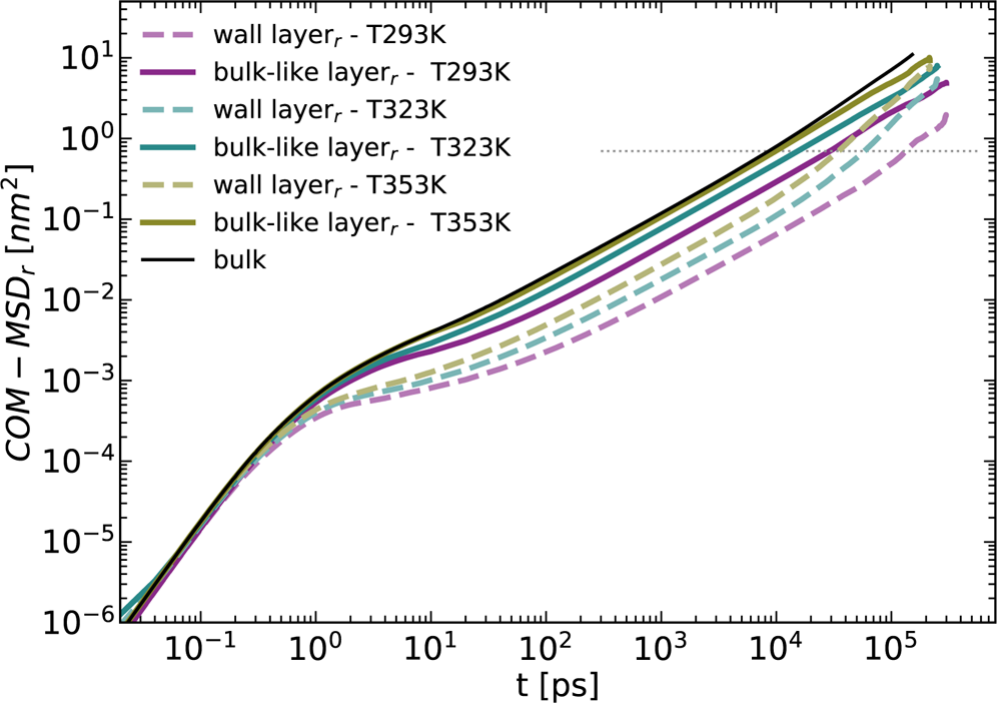
COM MSD in the melt along the radial direction for the layers closest and furthest away from the rod at three different temperatures. The dotted horizontal line is the value *R*_g_^2^/3 that we employ later to define the characteristic relaxation time.

**Figure 5:**
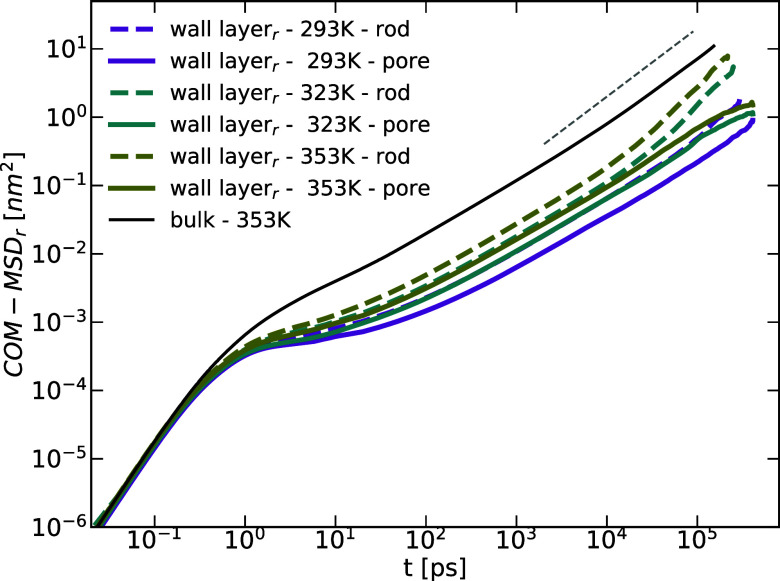
Comparison of the COM MSD (along the radial direction) for the wall (PBD–alumina interface) layer in both systems: solid lines for the nanopore and dashed lines for the nanorod system. The solid black line is the bulk COM MSD at *T* = 353 K and the dashed gray line shows *t*^1^ diffusive behavior.

Figure 4 shows the COM MSD along the radial direction for all three temperatures for the layer at the rod wall and for the farthest layer from the alumina. Following a ballistic regime, which remains unaffected by the proximity of the chains to the rod wall, the COM dynamics experience a slowdown at the interface. This slowdown affects the motion along the axial direction as well across all simulated temperatures as we will later see. Over longer time scales, the COM motion in the wall layer, particularly at higher temperatures, converges with that in the bulk-like layer. It is important to note that statistics at these extended time scales may not be as robust, but we do observe a consistent increase in displacement within the interface layer after a certain time, especially as the temperature increases. This suggests that with sufficiently long simulation times, the chains within the wall layer are likely to break free from it and exhibit behavior similar to that of the bulk (Figure 5).

To adopt a more quantitative approach, we define a characteristic relaxation time denoted as τ_R_, where  nm^2^. This value corresponds to the gray horizontal dashed line in Figure 4. We extract τ_R_ for all three layers in the melt, across all simulated temperatures, and list the corresponding values in Table 1.

**Table 1:** Characteristic Relaxation Time τ_R_ Extracted from COM MSD^a^

	τ_R_ [ps]					
	radial			axial		
	L1^b^	L2^c^	L3^d^	L1^b^	L2^c^	L3^d^
293 K	29,280	41,280	132,700	24,480	30,260	90,640
323 K	15,400	22,360	58,360	12,860	15,780	43,820
353 K	9860	14,280	34,820	8400	10,000	23,220

a The values correspond to the three different layers in the melt for both the axial and radial motion at *T* = 293, 323, and 353 K.

b L1 corresponds to the layer farthest away from the nanorod.

c L2 corresponds to the intermediate layer.

d L3 corresponds to the layer at the interface.

The τ_R_ values presented in Table 1 indicate a slowing down for the layers in proximity to the wall. These values are suitable for comparison with their counterparts from the nanopore system. Compared to the latter, τ_R_ (rod) shows faster dynamics and shorter relaxation times for all temperatures and layers. The characteristic time in bulk-like layers for the rod are comparable to those of bulk PBD with τ_R_ (bulk) = 9 ns vs τR__⊥__ (rod) = 9.8 ns and τR__∥__ (rod) = 8.4 ns.

The most notable and observable impact on the COM displacement occurs in the wall layer within the nanopore structure. However, this effect is reduced when the curvature is altered, as seen in the nanorod system. Unlike the pore system, where the melt is fully confined in two dimensions along the radial direction while free to diffuse in the axial direction, the rod system lacks this additional constraint on the chain motion.

**Figure 6:**
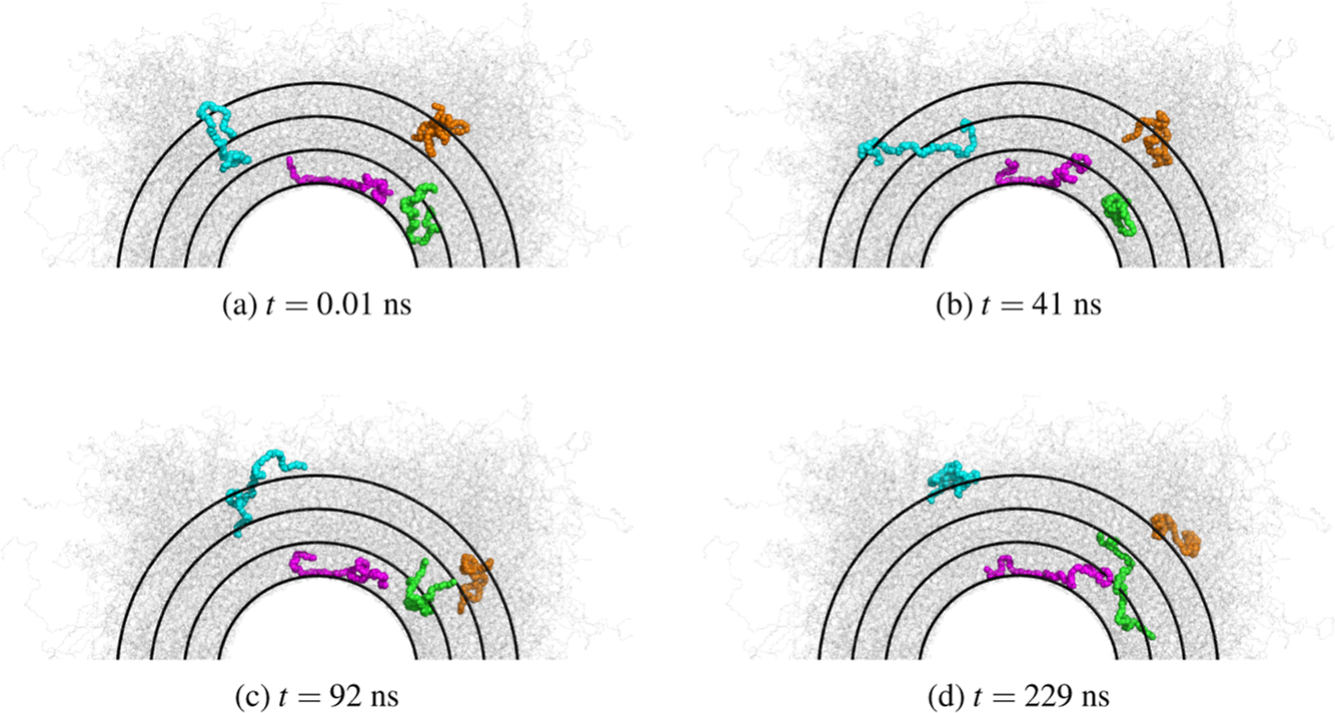
Snapshot of four different chains (green and magenta—chains 851 and 1170/cyan and orange—chains 586 and 674) at four different times along the simulation trajectory (*T* = 293 K). The black rings indicate the different layers of size ∼*R*_g_ starting with the smallest at the rod wall at a radial distance of 5 nm from the center of the rod.

To visualize the movement and configuration of the chains within the melt, we show in Figure 6 four distinct chains at *T* = 293 K over the course of the trajectory. The black circles denote various layers, which are used to calculate the dynamic properties. These layers begin with the innermost one located at a radial distance of 5 nm from the center of the rod. Two of the chains (green and magenta) initially have their COM within the first wall layer, while the other two chains (orange and cyan) start with their COM further away from the nanorod. One wall chain (magenta) begins with most of its monomers adsorbed to the surface, wrapping around the rod walls, whereas the other chain (green), although its COM is in the first layer, only has a few monomers directly at the interface. The magenta chain is stretched and oriented along the rod walls, and importantly, it remains adsorbed, indicating that its complete desorption process occurs on a time scale beyond that of this simulation. Such elongated and nearly fully adsorbed chains are relatively uncommon at the interface; nevertheless, studying their behavior holds significance. In contrast, the green chain, similar to most of the other chains in the first layer, undergoes the desorption process even at the lowest temperature and moves away, with its COM transitioning beyond the first wall layer.

#### Adsorption Autocorrelation Function

We have previously introduced the notion of monomer and chain adsorption onto the alumina walls. In the section discussing the melt’s structure, the density profile strongly suggests the existence of a layer of adsorbed monomers at the walls. The results derived from the analysis of the COM MSD reveal the impact of adsorption/desorption kinetics on the motion of the chains. Our definition of monomer adsorption is based on geometric criteria and is quantified by an adsorption autocorrelation function (ACF) Φ(*t*)
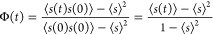
4

In eq 4, we define *s*(*t*) = 1 when the monomer is adsorbed at time *t*, and *s*(*t*) = 0 otherwise. To identify adsorbed monomers, we select chains whose centers of mass are within the wall layer of approximately size *R*_g_ at *t* = 0 (the length scale of COM density variation). Subsequently, adsorbed monomers are those belonging to these chains and positioned within the first layer at the wall, with a thickness of approximately 0.5 nm (roughly the monomer size). Consequently, *s*(0) = 1, leading to Φ(0) = 1. The decay of the correlation function over time provides insights into the time scale of monomer desorption. The comparison reveals that the initial desorption kinetics is essentially identical for the two geometries, i.e., it is determined by the interaction energy with the alumina walls. The long time kinetics, however, is different, because the desorption from the rod is into an open bulk, while that from the pore walls is into a confined system protracting the complete desorption of the chains.

### Dynamics on the Monomer Scale

#### Incoherent Neutron Scattering Function

To characterize the dynamics of the monomers in the melt we first resort to the incoherent neutron scattering function (INSF) *F*_s_(**q**,*t*). The latter provides us with information on the translation motion of the atoms in the system and is defined as
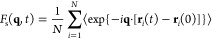
5where **r**_*i*_(*t*) is the position vector of the scattering center *i* at time *t* (the scattering centers in our system are the united atoms), **q** is the scattering wave vector also called the momentum transfer, *N* is the total number of atoms in the layer or the system and the angular brackets denote the thermodynamic average. The INSF for the confined systems is experimentally accessible and two different directions of the momentum transfer **q** can be considered: along  and along . To obtain that, the sample is rotated at 45° (momentum transfer perpendicular/along ) and 135° (momentum transfer parallel/along ) to the incident beam with a scattering angle of 90°.

**Figure 7:**
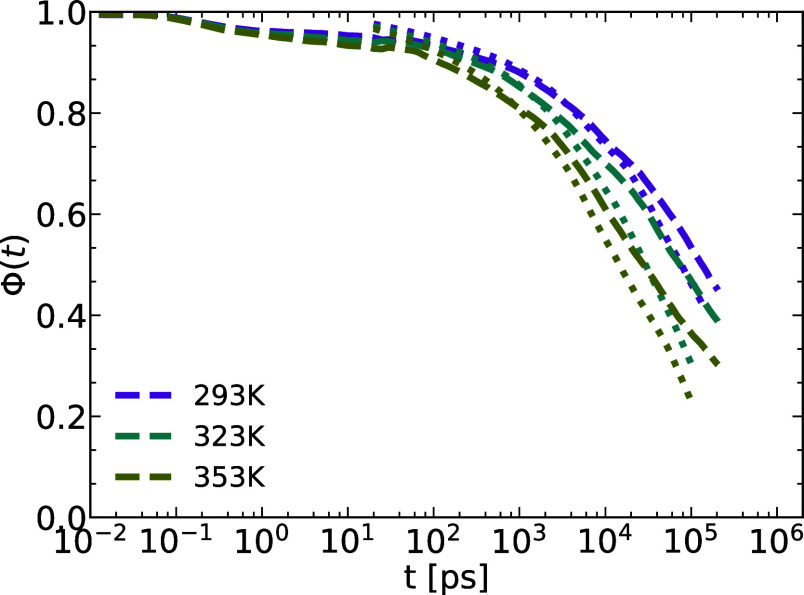
Adsorption ACF for the pore and the rod for the three indicated temperatures.

**Figure 8:**
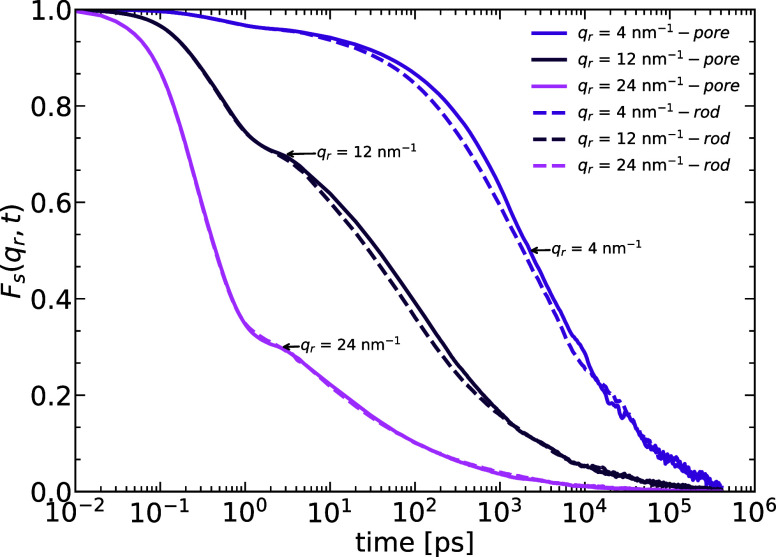
INSF for three different values of the momentum transfer **q**_*r*_ = 4, 12, and 24 nm^–1^ for the wall layer in both systems.

**Figure 9:**
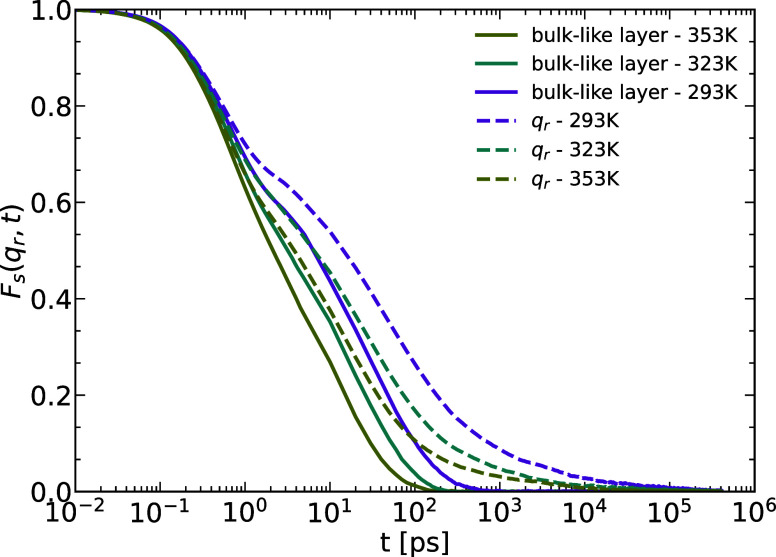
INSF calculated for the melt as a whole (dashed) and the wall layer (solid) in the nanopore system with **q**_*r*_ = 12 nm^–1^.

To study the motion along the radial direction of the melt in both systems, **q** should be perpendicular to the pore/rod axis. Averaging over all possible directions of the momentum transfer in the *xy*-plane, we obtain an expression of the *F*_s_(**q**_*r*_,*t*) = 2π/*NJ*_0_(**q**_*r*_*r*) in terms of the Bessel function of first kind *J*_0_. In Figure 8 the incoherent scattering is shown for three values of momentum transfer covering the range from very local to large scale motion. Locally, a clear plateau occurs in the scattering function, indicating the time scale separation between short time vibrations and long time relaxations. Since we are working far above the bulk transition temperature, this reveals the increased density in the adsorbed layers (density effect) as well as the slow desorption kinetics visible in Figure 7 (energetic effect). Both systems show basically the same behavior, with perhaps a slightly faster relaxation on the long length scales for the rod system. While the clear glass-like signature of plateau regimes is only observable for the wall layer and not for the bulk layer (as can be seen from the full lines in Figure 9) the scattering from the whole melt in the pore case has about equal contributions from the wall and the bulk region, so their average still shows a slowing down compared to the bulk dynamics even at temperatures far above the bulk *T*_g_ (Figure 9). This was fist reported from scattering experiments on Polyethylene-oxide confined in alumina pores.^65^

#### Spin–Lattice Relaxation

The local reorientation dynamics can be studied with spin–lattice relaxation nuclear magnetic resonances (NMR) experiments. These are measuring the reorientation of CH-vectors along the chain. We have shown for the bulk case that for PBD one can separately analyze the dynamics of different positions in the monomer.^66^ Here we will focus on the relaxation behavior of the CH vectors connecting the sp^2^ carbons in the trans and cis groups respectively to their attached hydrogen atoms. As we are using a united atom model for the simulation we have to reintroduce hydrogen atoms into stored configurations along the simulation trajectory following the procedure in ref (66). The second Legendre polynomial of the CH vector autocorrelation is given by
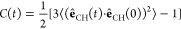
6The time integral of this ACF defines the correlation time
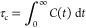
7The spectral density is the Fourier transform of the ACF
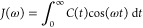
8And serves to determine the spin–lattice relaxation time
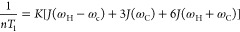
9Here ω_H_ and ω_C_ are the Larmor frequencies of hydrogen and carbon, respectively, and the number of bound hydrogens is *n* = 1 and *K* = 2.42 × 10^9^ s^–2^ for the sp^2^ carbon.

**Figure 10:**
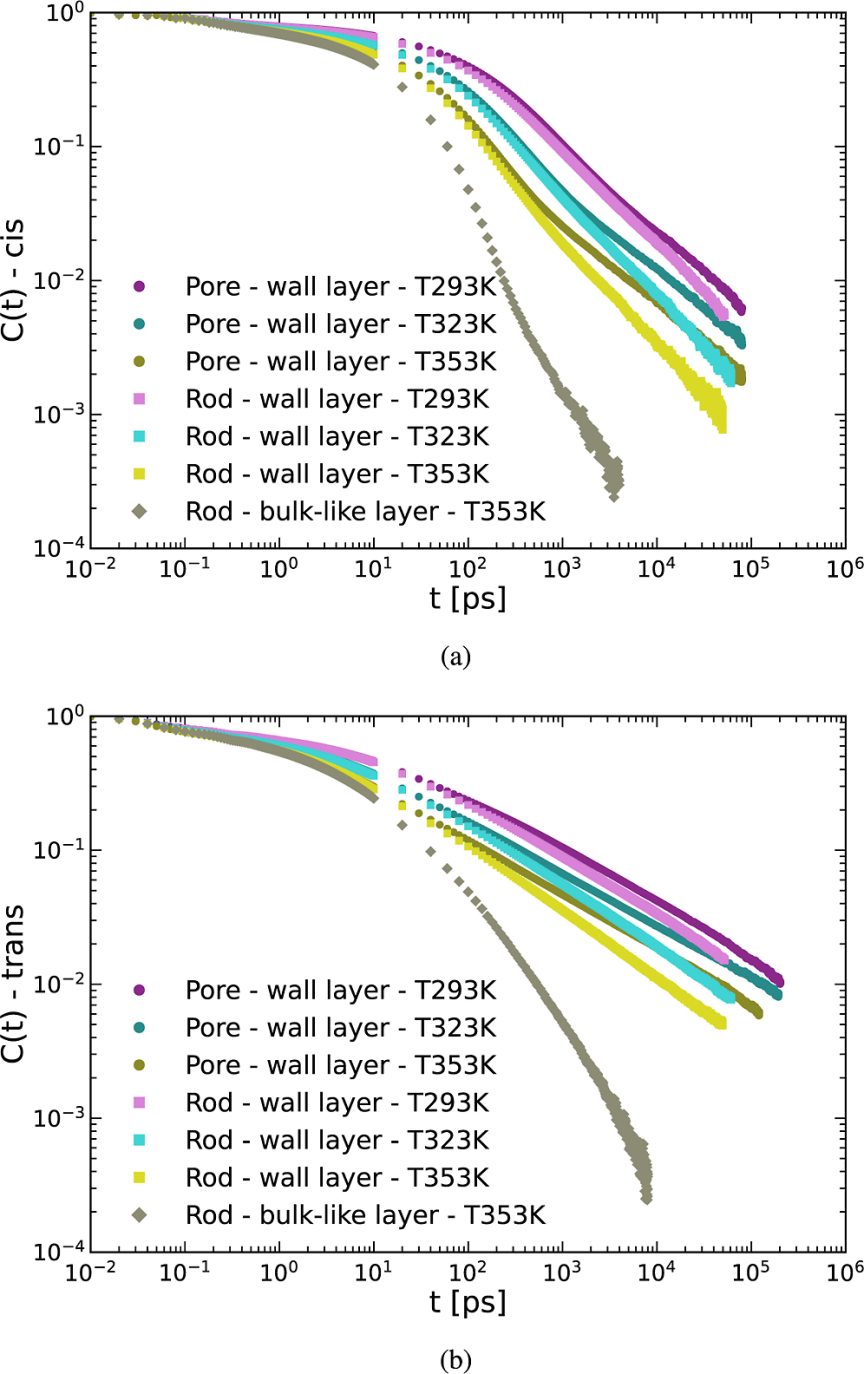
Orientation ACF for the cis (a) and trans (b) conformations. Both figures show the pore (circles) and rod (squares) wall layers for all three simulated temperatures as well as the bulk-like layer for the rod system at *T* = 353 K (gray diamonds).

In Figure 10 we compare the orientation autocorrelation functions of CH vectors in cis and trans groups in the wall layer for the pore and the rod system and the bulk behavior (for better visibility we show only the highest temperature for the bulk). Clearly, the relaxation in the wall layer is very similar for both geometries and strongly slowed down compared to the bulk relaxation. Again, the long time behavior for the rod system is faster than for the pore system for the mentioned reason. To quantify the change in relaxation time scales we calculate the correlation time as well as the spin–lattice relaxation time which are shown in Table 2 for the pore case and in Table 3 for the rod case. We also include bulk values from the literature.

**Table 2:** **Nanopore**: The Correlation Times τ_c_ (Top) and *T*_1_ (Bottom) Calculated from the C–H Bond Relaxation at Three Different Temperatures for Both the Cis and Trans Groups^a^

τ_c_ [ps]						
	trans			cis		
	nanopore			nanopore		
*T* [K]	center	wall	bulk	center	wall	bulk
293	171	20 919	305	111	3725	184
323	87	12,758	78	59	2148	61
353	46	9137	37	32	992	26

*nT*_1_ [s]						
	trans			cis		
	nanopore			nanopore		
*T* [K]	center	wall	bulk	center	wall	bulk
293	0.79	0.55	0.61	0.54	0.33	0.37
323	1.22	0.84	1.03	1.01	0.49	0.85
353	1.85	1.08	1.95	1.62	0.77	1.7

a The values for the bulk are taken from ref (66).

**Table 3:** **Nanorod**: The Correlation Times τ_c_ (Top) and *T*_1_ (Bottom) Calculated from the C–H Bond Relaxation at Three Different Temperatures for Both the Cis and Trans Groups^a^

τ_c_ [ps]						
	trans			cis		
	nanorod			nanorod		
*T* [K]	bulk-like	wall	bulk	bulk-like	wall	bulk
293	124	17 689	305	87	2446	184
323	65	5818	78	46	685	61
353	36	3064	37	27	341	26

*nT*_1_ [s]						
	trans			cis		
	nanorod			nanorod		
*T* [K]	bulk-like	wall	bulk	center	wall	bulk
293	0.85	0.56	0.61	0.66	0.35	0.37
323	1.45	0.85	1.03	1.15	0.53	0.85
353	2.11	1.08	1.95	1.83	0.81	1.7

a The values for the bulk are taken from ref (66).

## Conclusion

We have studied the confinement induced changes in structure and dynamics of a 1,4-polybutadiene melt in contact with an alumina surface. We compared the effects of confinement within an alumina pore to those induced by an alumina rod dispersed in the melt. Pore and rod had equal absolute curvature.

The largest influence both on statics and dynamics is induced by the strong attraction of the PBD monomers to the alumina surface. It leads to a significant layering in the monomer density as well as the COM density. The layering is stronger than in the case of PBD at a graphite wall, but most importantly, the strong attraction to the alumina has a different effect on the chain scale. Where the chains were only oriented but not deformed (as judged by their gyration ellipsoid) in the graphite case, the attraction to the alumina surface leads to a strong deformation of the chains in the vicinity of the surface, reminiscent of the adsorption transition of isolated chains at a surface. We find a broad distribution in the number of adsorbed monomers per chain, with a few chains having a macroscopic amount of monomers, *O*(*N*), adsorbed to the surfaces. The polymer material close to the surface therefore does not show the Gaussian chain statistics typical for a polymer melt.

Dynamically, the attraction leads to glass-like dynamics on the monomer scale as observable in incoherent scattering or NMR experiments, when one addresses only the relaxation in the wall layer. This occurs already in the high-temperature melt far above the bulk glass transition as it is governed by the energy scale of the polymer–wall interaction. Large scale and long-time relaxation within the wall layer is determined by the desorption process which possesses a time scale many times larger than the longest bulk relaxation time.

For our geometry, where the curvature radius is about three times the radius of gyration of the chains, we can not identify a significant effect of the absolute curvature; however, the sign of the curvature matters. In the rod geometry, the desorption process moves the chains into a free bulk melt, whereas in the pore geometry the chains desorb into a confined bulk. This makes the long time dynamics in the rod case faster then in the pore case.
